# Successful regenerative response of a severe bone defect in a right lower central incisor affected by a cemental tear

**DOI:** 10.1002/ccr3.6472

**Published:** 2022-11-05

**Authors:** Takayoshi Nagahara, Katsuhiro Takeda, Sadayuki Inoue, Keinoshin Wada, Hideki Shiba

**Affiliations:** ^1^ Nippon Kokan Fukuyama Hospital Hiroshima Japan; ^2^ Department of Biological Endodontics, Graduate School of Biomedical and Health Sciences Hiroshima University Hiroshima Japan; ^3^ Wada Dental Clinic Hiroshima Japan

**Keywords:** cemental tear, periodontal regeneration therapy, recombinant human fibroblast growth factor‐2

## Abstract

Cone‐beam computed tomography and clinical examinations including pulp vital testing and pocket probing depth showed a cemental tear with a severe labial alveolar bony defect, but no endodontic lesions, in #25, which had a sinus tract at the labial site, in a 75‐year‐old woman.

## Abstract 2

The present case report describes the clinical management of a cemental tear with severe periodontal tissue destruction that included complete removal of the torn fragments and periodontal regenerative treatment with novel therapy using rhFGF‐2.

## 1 INTRODUCTION

A cemental tear, the detachment of cementum from the root surface, may cause periodontal breakdown, although its pathogenesis has not been fully elucidated.^1^, ^2^, ^3^ A cemental tear often mimics periapical periodontitis, vertical root fracture, or an endodontic‐periodontal lesion.^2^, ^4^, ^5^, ^6^, ^7^, ^8^ Therefore, interpretation of the images of dental X‐rays and cone‐beam computed tomography (CBCT) and of clinical examinations, including pulp vital testing and measurement of periodontal pocket depth, needs to be carefully conducted to distinguish a cemental tear from the other lesions.^2^, ^4^, ^5^, ^6^, ^9^, ^10^, ^11^, ^12^

Our previous case involved a mandibular right central incisor affected by a perforation into a labial site and cemental tear that underwent an endodontic approach to perforation repair and periodontal treatment including periodontal regenerative therapy using recombinant human fibroblast growth factor‐2 (rhFGF‐2) to the labial bone defect.^13^ These treatments decreased pocket depth (PD), with a 5‐mm gain in the clinical attachment level (CAL) and proximal bone regeneration.^13^ However, sufficient reconstruction of the labial bone was not observed on cone‐beam computed tomography.^13^

Lee et al.^2^ have recently reported a new classification for cemental tears and recommendations for treatment strategies. The present case report shows that removal of torn cemental fractions and granulation tissues and the application of rhFGF‐2 in a mandibular right central lower incisor with a sinus tract, cemental tear, and severe localized periodontal destruction, which had been diagnosed as Class 3/Stage C based on the classification by Lee et al., led to significant clinical improvement (PD reduction, CAL gain, and bone regeneration).

## 2 CASE REPORT

**FIGURE 1:**
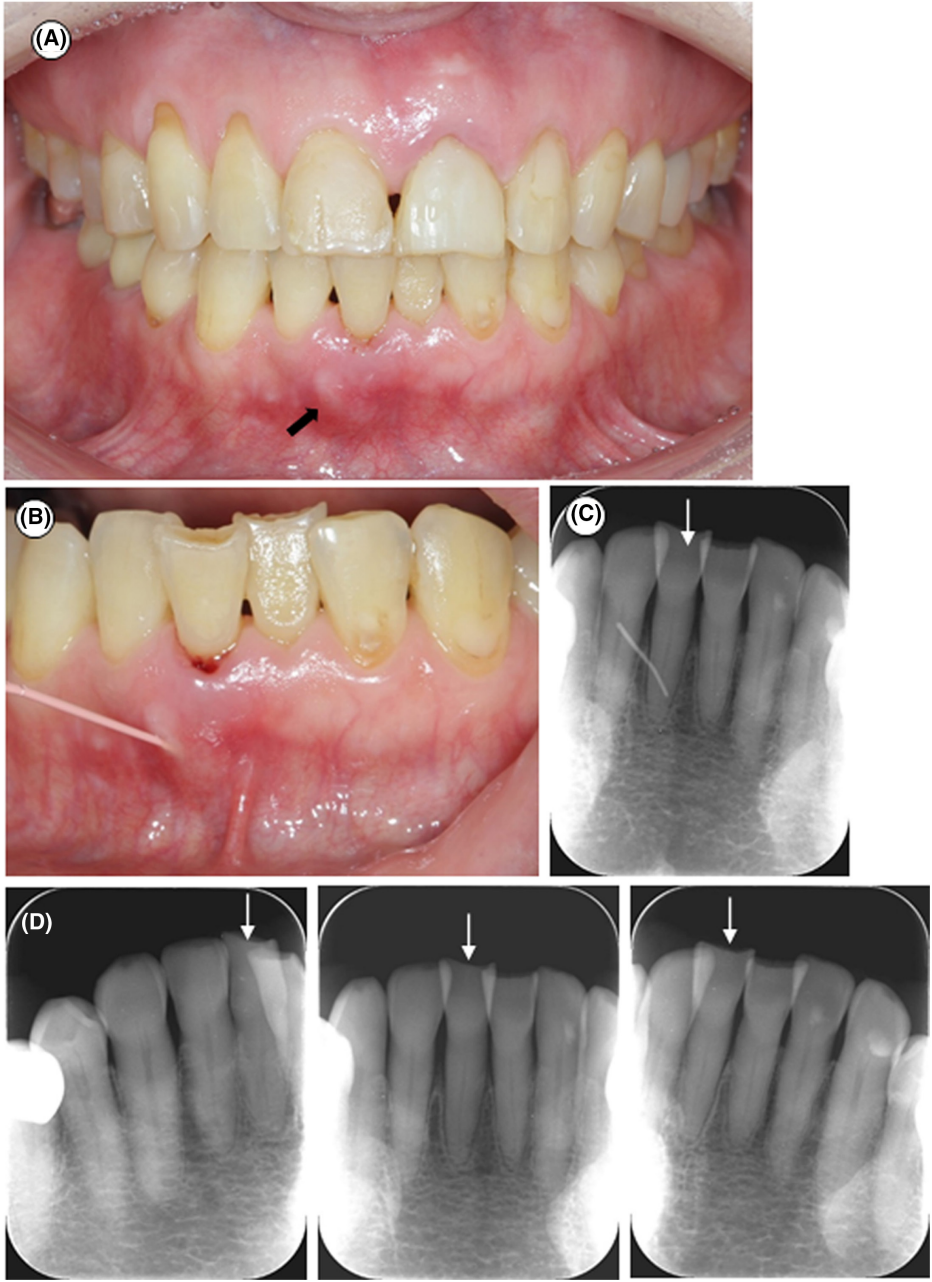
Intraoral photographs and dental radiographs at the first visit. (A) Intraoral photograph at the first visit. Redness and swelling are observed at the labial gingiva around #9 and #25. #25 has a sinus tract (black arrow). (B) Sinus tract tracing with a gutta‐percha point. (C) Radiographic view showing the gutta‐percha point from the sinus tract ending at one‐fourth of the root length from the root apex of #25. White arrow: #25. (D) Dental radiographs of the orthoradial projection and eccentric projection. No cemental tear is observed at the root of #25. White arrow: #25.

A 75‐year‐old woman with osteoporosis visited Nippon Kokan Fukuyama Hospital with a chief complaint of a gingival abscess and swelling around #9. Her gingiva in all areas of the mouth except #9 and #25 was healthy (Figure 1A), and she had good oral hygiene (plaque control record: 13.8%). Her #9 had a history of trauma 2 years earlier and was found to have a vertical root fracture. Therefore, #9 was extracted with her informed consent. In contrast, the patient had no subjective symptoms related to #25. A sinus tract was observed on the labial side of the gingiva around #25 (Figure 1A); #25 responded positively to thermal and electric pulp vital tests by PULPER® (GC, Tokyo, Japan) and Digitest® (Parkell, Farmingdale, NY, USA). Pocket probing depth (PPD) on #25 was 3 mm in all areas except the labial center, whose PPD was 9 mm. Probing of the labial center resulted in bleeding. Clinical attachment loss at #25 of the labial center, which was measured starting from its incisal edge, was 16 mm; #25 was inclined labially and showed tooth attrition (Figure 1B). A gutta‐percha point (size 40/02; GC Dental Industrial Corp., Tokyo, Japan) was used to trace the periodontal pocket of the labial center (Figure 1B). Radiography showed that the tip of the gutta‐percha point inserted into the sinus tract reached the mesial site at one‐fourth of the root length from the root apex of #25 (Figure 1C). Radiolucent areas were not observed around the tip of the inserted gutta‐percha point (Figure 1C). Dental radiographs in the orthoradial projection and the eccentric projection of #25 showed no radiopaque fragments at the root (Figure 1D). The patient's oral hygiene was good, and her gingiva and bone levels in all areas of the mouth except #25 were healthy, suggesting that #25 should be classified as a periodontal abscess in a non‐periodontitis patient.^14^, ^15^

**FIGURE 2:**
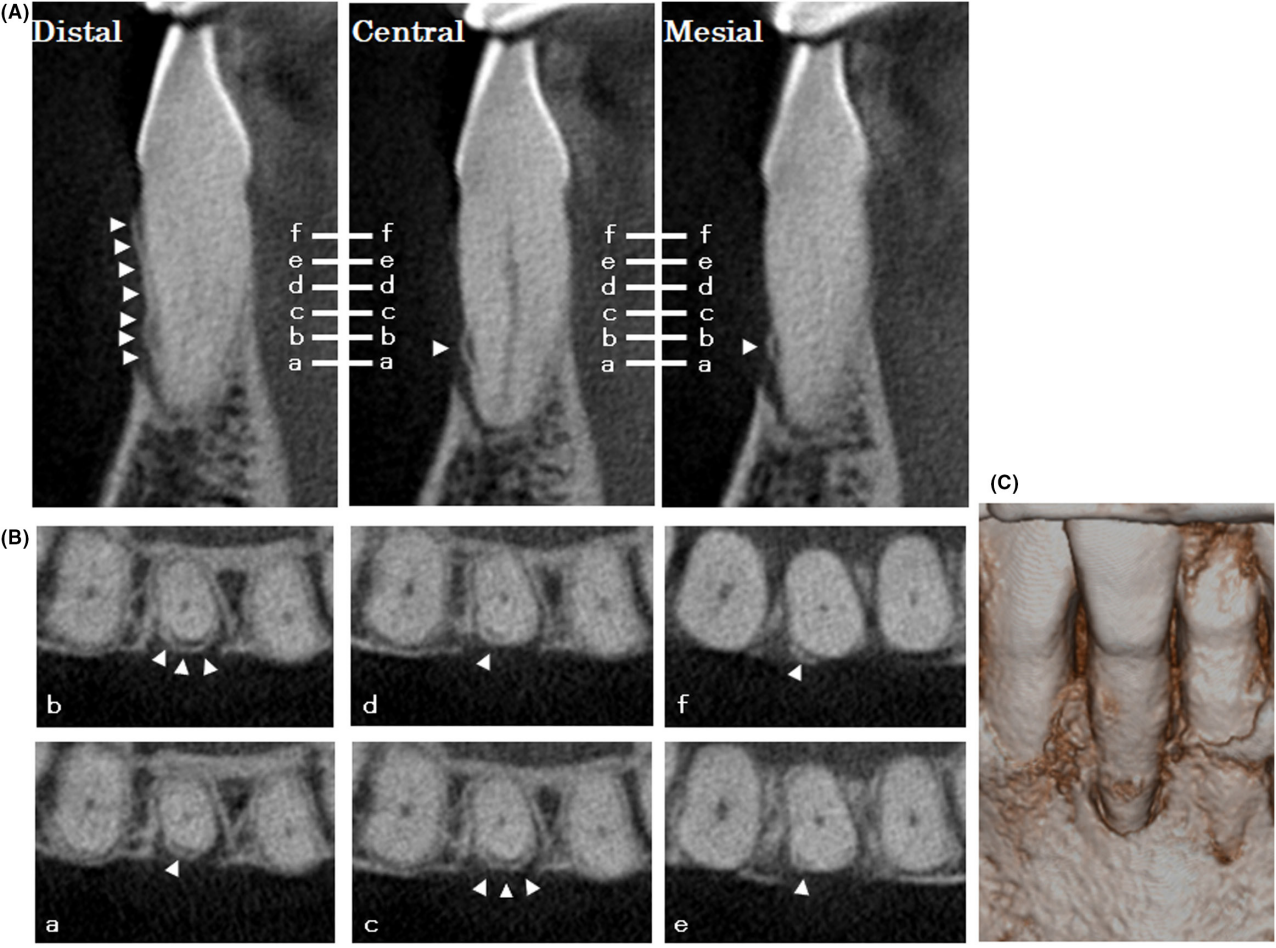
CBCT images of #25 at the first visit. (A) Sagittal reconstructed CBCT images at the point of the distal, central, and mesial sides. Arrowhead: cemental tear. (B) Coronal reconstructed CBCT images at the point of a‐f slice lines in (A). Arrowhead: cemental tear. (C) Three‐dimensional reconstruction of CBCT images.

Cone‐beam computed tomography (CBCT) images (3DX Multi‐Image Micro CT FPD8; J Morita, Tokyo, Japan) of #25 showed an extensive bone defect on the labial aspect and narrow‐range defects on the mesial and distal aspects at the buccal side (Figure 2A,B). Three‐dimensional reconstruction of CBCT images showed narrow, shallow, vertical bone defects from the existing marginal bone in the three sites (Figure 2C). Radiopaque thin fragments, which were completely and incompletely detached from the root surface, were observed at the distal‐labial and mesial‐labial aspects of the root (Figure 2A,B). This was diagnosed as a case of cemental tear accompanied by severe periodontitis. According to the new three‐dimensional classification of cemental tears,^2^ this case was classified as Class 3 (clinically inaccessible, infrabony and/or dehiscence, no apical involvement)/Stage C (cemental tear and the associated bony defect involves 3 surfaces of the root). The clinical symptoms and the results of the examinations suggested periodontal treatment (surgical removal of cemental fragments and granulomatous tissue, biopsy of removed tissues, and periodontal regenerative therapy with rhFGF‐2), but not endodontic treatment. Informed consent was obtained from the patient after explanation of the risks, benefits, and costs of the proposed treatments.

**FIGURE 3:**
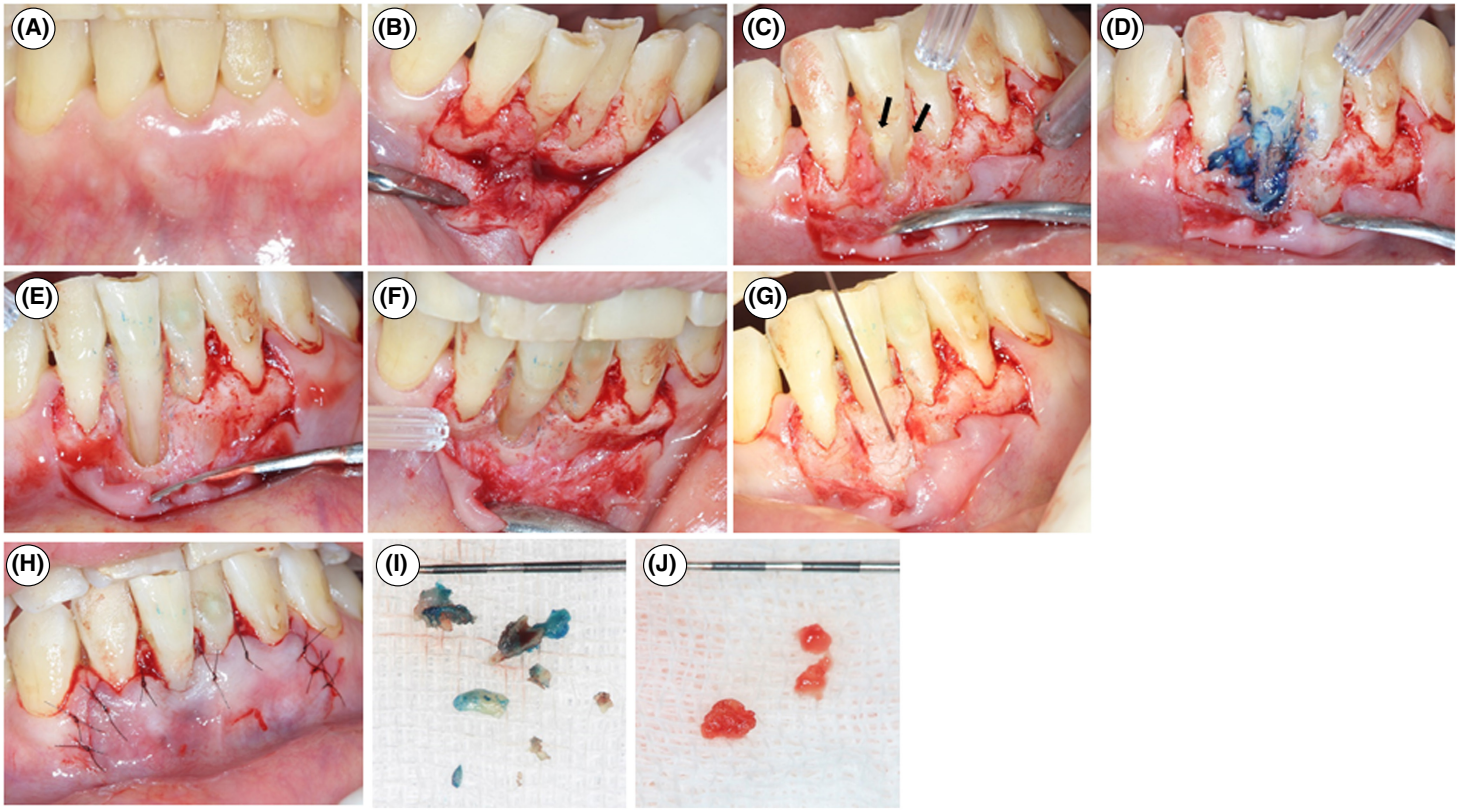
Periodontal regenerative therapy with rhFGF‐2. (A) Presurgical view. (B) Single‐flap approach with labial access. (C) After removal of granulomatous tissue. Cemental fragments (black arrows) are observed. (D) Methylene blue dye staining shows the cemental tear clearly. Then, granulomatous tissue is completely removed by the curette. (E) Labial view after the removal of the cemental fragment. (F) Occlusal view after the removal of the cemental fragment. (G) Application of rhFGF‐2. (H) After suture. (I) Removed cemental fragments. (J) Removed granulomatous tissues.

Occlusal adjustment was conducted at the anterior tooth to improve occlusal function after extraction of #9. After scaling and root planing, the periodontal pocket (PPD of the labial center: 9 mm) remained, although the swelling, abscess, and sinus tract had disappeared (Figure 3A). With disinfection by povidone iodine and local anesthetic administration with Xylocaine® (DENTSPLY‐Sankin Co., Tochigi, Japan) in the right mandibular region, a full‐thickness mucoperiosteal flap with a single‐flap approach to the labial access was raised (Figure 3B). After granulomatous tissue was partially removed (Figure 3C), cemental fragments on the root were stained with methylene blue dye (Morimura Dental Co., Tokyo, Japan) (Figure 3D). After the removal of cemental fragments and the remaining granulomatous tissue with hand curettes under the dental operating microscope, the range of the bone defect was found on the proximal and labial aspects (Figure 3E, F). Subsequently, root planing was conducted. A thin dentin defect that did not extend to the wedge‐shaped defect occurred on the labial‐site root. For this, rhFGF‐2 (Kaken Pharmaceutical Co., Tokyo, Japan) was applied into the bone defect (Figure 3G). The flap was then repositioned without tension and sutured with 7–0 nylon (Mani, Tochigi, Japan) (Figure 3H). During surgery, the cemental fragments (Figure 3I) and granulomatous tissues (Figure 3J) were collected for biopsy.

**FIGURE 4:**
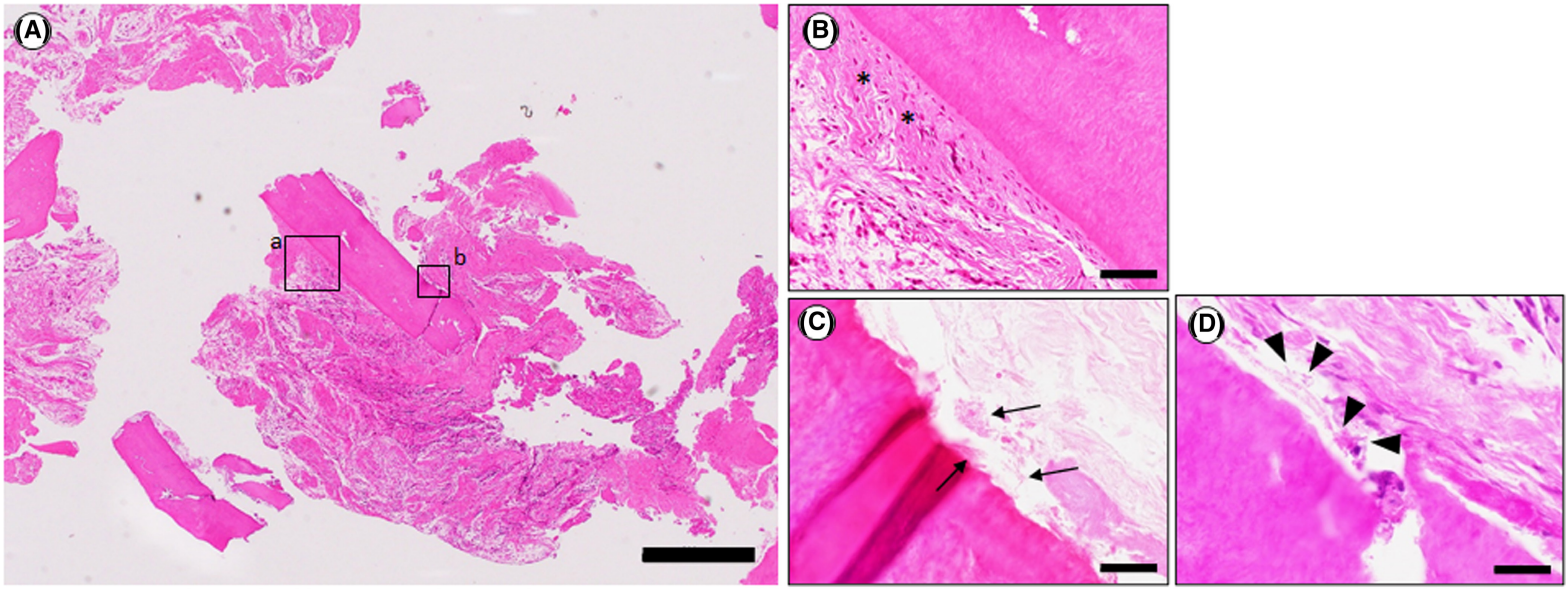
Histological examination of the removed cemental fragments. (A) A low‐power view of a cemental fragment shows the presence of mainly acellular cementum. HE staining. Scale bar: 500 μm. (B) Higher‐power view of the open square (a) in (A). Periodontal fibrous connective tissue (asterisk) is observed on the cemental fragment. HE staining. Scale bar: 50 μm. (C) Higher‐power view of the open square (b) in (A). Gram‐positive bacterial colonies are observed (black arrows). Gram staining. Scale bar: 20 μm. (D) Higher‐power view of the open square (b) in (A). PAS‐positive bacterial colonies are observed (black arrowheads). PAS staining. Scale bar: 20 μm.

**FIGURE 5:**
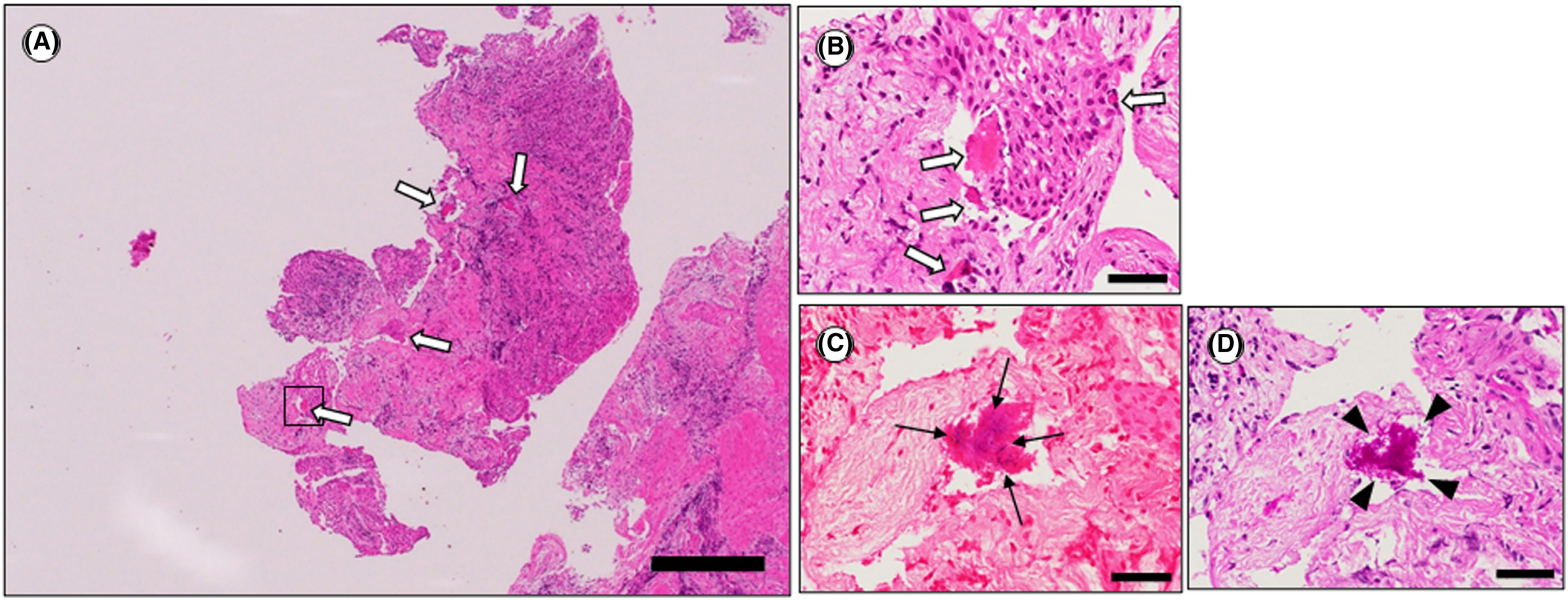
Histological examination of the removed granulomatous tissue. (A) A low‐power view of the granulomatous tissue. Dense fibrous tissue contains small, scattered cemental fragments (white arrows). HE staining. Scale bar: 500 μm. (B) Higher‐power view of the open square in (A). Cemental fragments within removed granulomatous tissue (white arrows). HE staining. Scale bar: 50 μm. (C) Higher‐power view of the open square in (A). Gram‐positive bacterial colonies are observed within the cemental fragment (black arrows). Gram staining. Scale bar: 20 μm. (D) Higher‐power view of the open square in (A). PAS‐positive bacterial colonies are observed within the cemental fragment (black arrowheads). PAS staining. Scale bar: 20 μm.

Histopathological examination of the removed cemental fragment with hematoxylin and eosin (HE) staining showed the acellular cementum (Figure 4A, B). A thin layer of fibrous connective tissue representing the periodontal ligament was attached to the cemental fragment (Figure 4B). Gram‐positive bacteria (Figure 4C) and periodic acid‐Schiff (PAS)‐positive bacteria (Figure 4D) were observed on the cemental fragment. Small, scattered cemental fragments were present within the removed granulomatous tissue (Figure 5A, B). Gram‐positive bacteria (Figure 5C) and PAS‐positive bacteria (Figure 5D) were observed on the small, scattered cemental tears within the granulomatous tissue. These findings support the previous report that bacteria invade the concealed site of the fractured fragments of a cemental tear and scattered cemental fragments within the granulomatous tissues to colonize, proliferate, and survive.^13^

**FIGURE 6:**
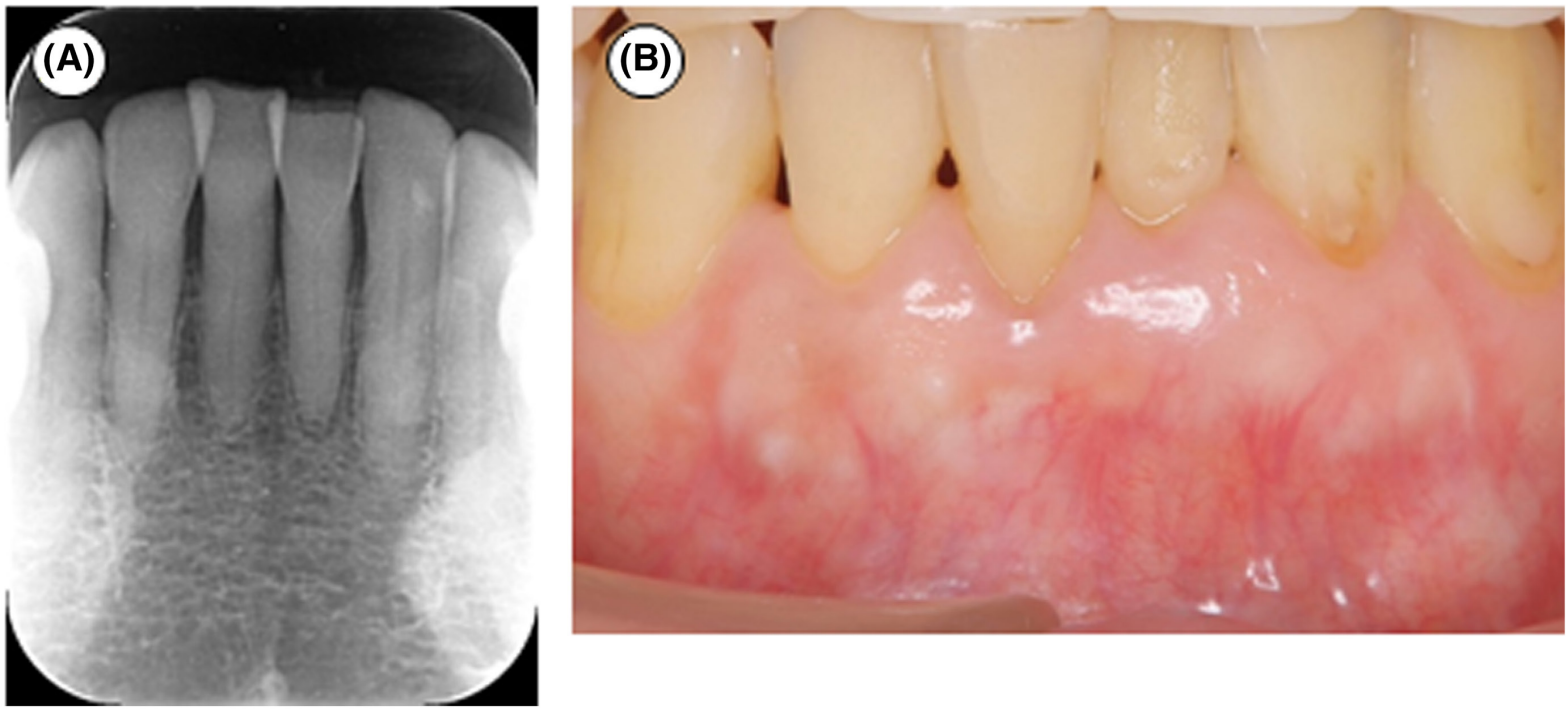
Dental radiograph and intraoral photograph of #25 two years after the surgery. (A) Dental radiograph. No abnormal findings. (B) Intraoral photograph. Clinically healthy soft tissue.

**FIGURE 7:**
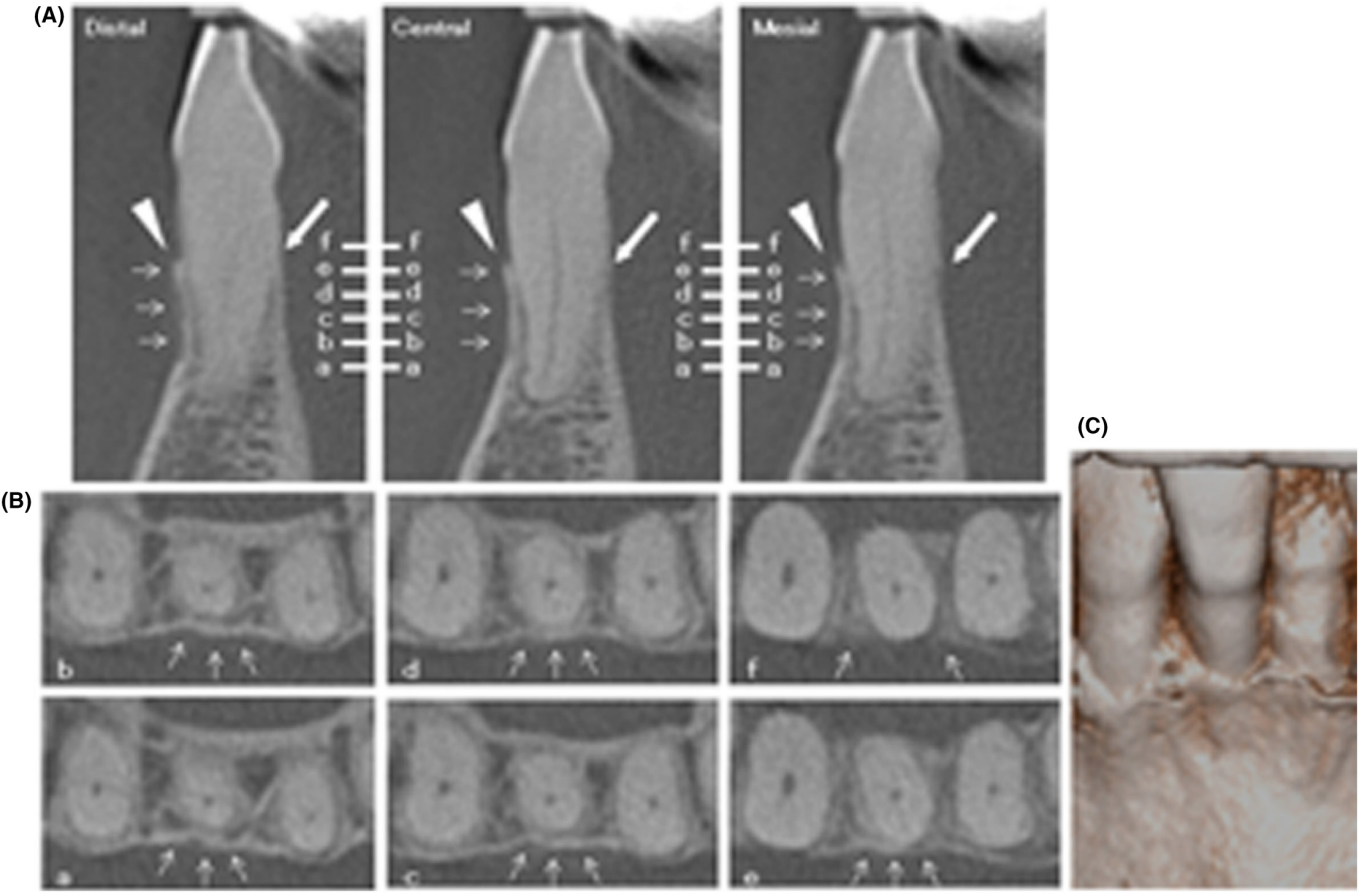
CBCT images of #25 2 years after the surgery. (A) Sagittal reconstructed CBCT images at the point of the distal, central, and mesial sides. Small arrows: bone regeneration. Arrowheads: labial bone level. Large arrows, lingual bone level. (B) Coronal reconstructed CBCT images at the point of a‐f slice lines in (A). Small arrows: reconstruction of the proximal bone and the labial bone. (C) Three‐dimensional reconstruction of CBCT images.

Two years after the surgery, no abnormal findings were seen radiographically (Figure 6A), and clinically healthy soft tissues were observed without severe gingival recession (Figure 6B). PPD of all sites was 2 mm. #25 had no bleeding on probing and no tooth mobility. The clinical attachment level of the labial center was improved from 16 mm to 9 mm (attachment gain: 7 mm). CBCT 2 years after the surgery showed reconstruction of the labial bone wall (Figure 7A–C) and proximal bone walls (Figure 7B). In particular, the regenerated labial bone had a similar height to the lingual bone (Figure 7A).

## 3 DISCUSSION

Although #25 had 9‐mm PPD in its labial center and a sinus tract at the labial gingiva, its dental radiograph showed no radiolucent area around the tooth root. CBCT images were obtained for examination of the presence or absence of lesions in the labial and lingual areas of the tooth root after the necessity of root canal treatment had been ruled out by pulp vital testing, which resulted in early diagnosis of a cemental tear with severe periodontitis of #25, and the clinical outcome was improved. In addition, the patient had healthy periodontal tissue, supporting the hypothesis that a cemental tear is one of the causes of a periodontal abscess in non‐periodontitis patients.^14^, ^15^

According to the new classification and treatment decision‐making, combined regenerative approaches using combinations of two or three of the biologic regenerative factors, bone replacement grafts, and collagen membrane barriers are recommended for Classes 3, 4, 5, and 6 and Stages B, C, and D with a 1‐, 2‐, and wide 3‐walled intrabony defect.^2^ Each simple regenerative approach is recommended for Classes 3 and 5 and Stage A with a narrow 3‐walled intrabony defect or crater.^2^ In fact, a case of treatment for cemental tear of Class 6/Stage C, for which the combined approach of application of enamel matrix derivative (EMD) and placement of Bio‐Oss was applied, has been reported.^2^ Thus, combined regenerative treatment is, in general, recommended in Class 3/Stage C‐case instead of a simple regenerative treatment. However, single regenerative treatment with a biologic regenerative factor was chosen in the present case of Class 3/Stage C because #25 had extensive labial bone defect with the labial center deep periodontal pocket.

Currently, rhFGF‐2 and EMD, which are approved as a drug and a medical device, respectively, under the Pharmaceuticals and Medical Devices Act are available as biologic regenerative factors for periodontal tissue regeneration in Japan. In the present case, rhFGF‐2, which can effectively regenerate periodontal tissue in patients who suffer from periodontal disease,^16^, ^17^, ^18^ was used because it has been reported that the efficacy of periodontal regeneration in an intrabony defect is superior with rhFGF‐2 compared to EMD.^16^

## 4 CONCLUSION

CBCT and clinical examinations including pulp vital testing and PPD are very important tools for evaluating periodontitis and/or endodontic lesions with a cemental tear located at labial/buccal and lingual/palatal sites. Complete removal of cemental fragments and periodontal regenerative treatment are indispensable clinical treatments for cemental tears with severe periodontal tissue destruction, although the size of the defect on root dentin after removal of cemental fragments and root planing in addition to the categories of class and stage (Lee et al.^2^) might be key factors that affect periodontal tissue regeneration.

## AUTHOR CONTRIBUTIONS

TN, KT, SI, KW, and HS: drafted the manuscript and contributed to treatment of the patient. All authors have read and approved the final manuscript.

## FUNDING INFORMATION

Public Expense of Hiroshima University (No.306100).

## CONFLICT OF INTEREST

The authors have no conflicts of interest to disclose.

## CONSENT

Written, informed consent was obtained from the patient to publish this report in accordance with the journal's patient consent policy.

## Data Availability

Data sharing is not applicable to this article as no new data were created or analyzed in this study.
